# Draft genome sequences of two strains of a putative novel *Bartonella* species isolated from a rodent and a flea in Chile

**DOI:** 10.1128/mra.00339-26

**Published:** 2026-06-15

**Authors:** Matías Goddard, Paulina Sepúlveda-García, Pablo Oyarzún-Ruíz, Macarena Nuñez, Lucila Moreno, Ma. Carolina Silva-de la Fuente, Luis Collado, Jaime Rodriguez, Armin Mella

**Affiliations:** 1 Instituto de Bioquímica y Microbiología, Facultad de Ciencias, Universidad Austral de Chile 28040, Valdivia, Chile; 2 Departamento de Microbiología, Facultad de Ciencias Biológicas, Universidad de Concepción 28056, Concepción, Chile; 3 Departamento de Zoología, Facultad de Ciencias Naturales y Oceanográficas, Universidad de Concepción 28056, Concepción, Chile; 4 Centro Nacional de Investigación en Ríos, Invasiones y Sistemas (IRIS)-CIN250047, Concepción, Chile; 5 Escuela de Medicina Veterinaria, Departamento de Ciencias Agrarias, Facultad de Ciencias Agrarias y Forestales, Universidad Católica del Maule, Curicó, Chile; 6 Facultad de Medicina Veterinaria y Agronomía, Universidad UTE, Santo Domingo, Ecuador; Indiana University Bloomington, Bloomington, Indiana, USA

**Keywords:** *Bartonella* spp., rodent-associated *Bartonella*, rodent, isolation, novel species

## Abstract

We report the draft genome sequences of *Bartonella* sp. nov., strains EBT8 and P12B, isolated from a rodent and a rodent flea in Laguna Verde, Chile. The genome assemblies showed values below the cutoffs for species definition (ANI values ≤ 95% and dDDH values ≤ 70%) against other *Bartonella* species, indicating that the strains represent a putative novel species of the genus *Bartonella*.

## ANNOUNCEMENT

*Bartonella* spp. are fastidious Gram-negative vector-borne bacteria, with numerous new species reported in recent years (1). Rodents act as their principal reservoirs, and rodent fleas efficiently transmit these bacteria (1). Several rodent-associated *Bartonella* species have been linked to human disease worldwide (2). In Chile, molecular surveys have suggested the presence of both known and potentially novel *Bartonella* species, but the lack of bacterial isolates has hindered confirmation (3–5).

The present study aimed to elucidate *Bartonella* species diversity associated with rodents and their fleas collected in Laguna Verde, Biobío region, Chile (36°47′38.1″S 73°09′33.0″W), in December 2024 and January, May, and December 2025. Animal procedures were authorized by the Chilean Agricultural and Livestock Service (SAG) under Exempt Resolution No. 2485/2023. Blood samples of captured rodents (*n* = 25) and fleas (*n* = 14) parasitizing them were cultured (6, 7). *Bartonella*-like colonies were confirmed by nuoG-qPCR (8). DNA was purified from colonies using the QIAGEN DNeasy Blood & Tissue Kit (QIAGEN, Hilden, Germany). For species identification, DNA was amplified for the *gltA* (9) and *rpoB* (10) partial genes and sequenced by Sanger method. BLASTn and Bayesian phylogenetic analysis evidenced that two strains, one from an *Oligoryzomys longicaudatus* rodent (EBT8) and another from a *Neotyphloceras* sp. flea (P12B), presented high identity and were phylogenetically closely positioned to an uncharacterized *Bartonella* previously detected in a Chilean rodent flea (PP151230, PP151224). These findings suggest the presence of a putative novel *Bartonella* species, prompting whole-genome sequencing for further characterization.

Genome sequencing was conducted at SeqCenter (Pittsburgh, PA, USA). Library was prepared using the Illumina DNA Prep kit and sequenced in a NovaSeq X Plus sequencer, generating 2 × 151 bp paired-end reads. Default parameters were used for all software, unless otherwise specified. Reads quality was assessed with FastQC v0.11.5 (11), and low-quality reads were removed with Trimmomatic v.0.39 (12). A *de novo* assembly was generated using Unicycler v0.4.8 (13), excluding contigs smaller than 1,000 bp. Assembly statistics were calculated with QUAST v5.0.2 (14); completeness and contamination was assessed with CheckM v1.2.4 (15). Annotation was performed during sequence submission to the NCBI using the Prokaryotic Genome Annotation Pipeline (PGAP) v6.10 (16). Average nucleotide identity (ANI) based on BLAST+ (ANIb) and digital DNA-DNA hybridization (dDDH) was calculated with JSpecies Web Server (17) and the Genome-to-Genome Distance Calculator (18), respectively, against the genomes of the validated species of *Bartonella* up to date (LPSN) (19)

A total of 13,933,158 and 11,754,122 reads were generated for EBT8 and P12B, respectively. EBT8 reads were assembled into 89 contigs with 985× coverage and an N50 of 195,254 bp. The genome is 2,031,139 bp with a G + C content of 38.47%, 1.849 genes, and 1.725 protein-coding sequences. P12B reads were assembled into 61 contigs with 869× coverage and an N50 of 182,346 bp. Its genome is 1,940,338 bp with 38.33% G + C, 1.731 genes, and 1.609 protein-coding sequences. Both assemblies have a completeness of 99.8% and 0.1% of contamination. The ANI between strains is 98%, and the dDDH is 89%. The ANI value supports their classification within the same species (ANI ≥ 96%); however, based on the proposed ANI thresholds for strain delineation (ANI ≥ 99.99%), they represent different strains rather than the same clone (20). The strains have ANI values ≤ 95% and dDDH values ≤ 70% against other validly published *Bartonella* species, values below the cutoffs for species definition (21), indicating that these strains represent a putative novel *Bartonella* species.

## Data Availability

All data are available under BioProject accession number PRJNA1370954. The raw reads were deposited at the SRA under accession number SRP651188. The GenBank genome accession number for the EBT8 strain is JBTTDJ000000000, and for the P12B strain is JBTTDI000000000. The version described here is the first version.
